# Dynamic Nurse Staffing for Better Accompanying Services in Outpatient Departments: Insight from West China Hospital

**DOI:** 10.3390/healthcare13080867

**Published:** 2025-04-10

**Authors:** Wei Wu, Zhoutianqi Yu, Maolin Zhuo, Li Luo, Qingyi Wang

**Affiliations:** 1 Department of Otolaryngology Head and Neck Surgery, West China Hospital, Sichuan University, Chengdu 610041, China; wuwei@wchscu.cn; 2 Outpatient Department, West China Hospital, Sichuan University, Chengdu 610041, China; 3 Business School, Sichuan University, Chengdu 610064, China; 2019141080027@stu.scu.edu.cn (Z.Y.); luolicc@scu.edu.cn (L.L.); 4 Faculty of Economics and Management, Universiti Kebangsaan Malaysia, UKM Bangi, 43600 Selangor, Malaysia; p146368@siswa.ukm.edu.my; 5 School of Finance and Trade Management, Chengdu Industry & Trade College, Chengdu 611731, China

**Keywords:** dynamic nurse staffing, accompanying services, outpatient departments, multi-period planning

## Abstract

**Background**: Over the past decade, the demand for accompanying services, which can be provided by both professional nurses and unskilled workers in major general hospitals, escalates significantly. Although nurses can provide higher value accompanying services to outpatients, their accompanying service capacity is limited and time-varied, which calls for the optimization of dynamic nurse staffing, an issue rarely addressed in the literature. **Objective**: To fill the gap, this study proposes a multi-period planning model for dynamically managing the staffing of nurses and workers for better delivering accompanying services in outpatient departments. **Method**: This model considers the dynamic arrival of various types of outpatients over a planning horizon and the coordination between nurses and workers in providing accompanying services with a goal of maximizing the value of accompanying services created for outpatients. Several comparison cases are investigated to highlight the benefits of our dynamic nurse staffing model. **Result and Conclusion**: With a case study on the outpatient department of West China Hospital, a representative large-scale general hospital in China, we demonstrate the effectiveness of the proposed model and generate managerial insights, which emphasize the dynamic and integral management of the staffing of nurses and workers to provide better accompanying services.

## 1. Introduction and Literature Review

The World Health Organization reports that the global healthcare market has an average annual growth rate of 16.4%, with a market size of approximately USD 0.83 trillion. In particular, the demand for outpatient services grows rapidly in countries with a large population such as China and India. Outpatient services are medical care (e.g., diagnosis, treatment, follow-up care, preventive health measures, and minor procedures) provided to patients who do not require overnight hospitalization. Due to factors such as aging population, healthcare system complexity, limited transportation and accessibility, and time constraints, the number of outpatients who need high-quality outpatient accompanying services has also increased quickly in recent years [1]. Outpatient accompanying services are professional or non-professional assistance provided to outpatients during medical visits, typically with the goal of ensuring that outpatients receive timely, organized, and effective care, particularly for those who face challenges due to age, disability, language barriers, or lack of medical knowledge. The accompanying services developed and provided by a hospital can be free or reasonably priced. Some typical accompanying duties include logistical support, physical accompaniment, communication facilitation, documentation aid, and emotional support. Nurses are an important but limited healthcare human resource, not only essential to meet the nursing demand of patients in normal situations but also important for response in emergency situations [2,3,4,5]. However, in many densely populated developing countries, such as India, Vietnam, and China, the number of nurses in hospitals is still limited, and nurses must assume multiple responsibilities and take care of many patients simultaneously. For example, in large general hospitals in China, a nurse takes care of eight patients during the day and twenty-three patients at night on average [6], indicating a much higher nursing workload.

To improve the medical experience of patients, some hospitals let nurses provide professional accompanying services to patients who find it difficult to seek medical treatment independently, but this has increased the burden and pressure on hospital nursing staff [7,8]. The increased nurse demand for accompanying services in outpatient departments can exacerbate the shortage of nurse resources. Thus, some large general hospitals let unskilled workers (e.g., volunteers and medical guides) provide accompanying services to reduce the workload of nurses [9]. For example, at West China Hospital, a representative general hospital in China, the number of outpatient and emergency visits in 2024 was about 8.42 million, and the outpatient accompanying service demand increased rapidly in recent years. To better meet the demand for accompanying services with rather limited nurse resources, West China Hospital forms a large human resource pool of unskilled workers and allows workers to provide basic accompanying services, such as assisting with registration, payment, and navigating hospital procedures, and it also requires certain numbers of nurses in the outpatient department to provide more professional accompanying services without compromising professional nursing services. Such field practices suggest that how to dynamically coordinate workers and nurses to better satisfy the demand for outpatient accompanying services is a key practical issue that needs to be addressed.

Although workers can provide basic accompanying services to outpatients and help to alleviate the shortage of nurse resources [10], most outpatients still prefer professional accompanying services provided by nurses. In contrast to the fact that workers typically lack sufficient medical knowledge and training to provide effective psychological and professional support to outpatients, nurses can provide better guidance and support to outpatients with their vast medical knowledge and nursing experience [11]. Given the dynamic fluctuations in the demand for general nursing services and accompanying services in the outpatient departments of large general hospitals, the value of accompanying services can be increased through the efficient and effective allocation and coordination of nurses and workers. Inspired by the field practices at West China Hospital during peak periods with high demand for general nursing services, nurses can focus on professional nursing work, and outpatient accompanying work can be performed primarily by workers. During periods with less general nursing demand, the staffing of the nurses can be adjusted to allow some nurses to provide outpatients with higher-quality accompanying services. However, it is a challenging task to develop a dynamic nursing staffing plan that increases the value of the accompanying services without greatly compromising the time-varied general nursing services.

In the literature, studies on nurse resource planning can be classified into two groups, i.e., static nurse resource management [12,13] and dynamic nurse resource management [14,15]. The key of static nursing resource planning is to achieve a reasonable allocation of resources based on the specific needs of the hospital in various periods and the professional service capacity of nurses. Rachaniotis et al. [16] point out that, in establishing a deterministic nurse resource planning model, it is necessary to comprehensively consider various factors to maximize the efficiency of nurse resource allocation. Yin et al. [17] use economic research methods to calculate the manpower demand gap of community health services in Xicheng District, Beijing, and thus propose a corresponding allocation plan. Myszewski and Sinha [18] optimize the quality of medical services by building a value model based on medical and health gaps, taking into account cost-effectiveness while maximizing patient satisfaction and value. In summary, the existing static research focuses more on identifying the allocation problems of nursing resources and formulating staffing strategies based on them, and maintaining relative stability over a certain period of time. However, given the complexity and dynamic nature of nursing human resource allocation, most studies continue to focus on exploring dynamic strategies for optimizing nurse resource allocation in multi-period situations to achieve more flexible and efficient resource allocation [19,20]. Bagheri et al. [21] propose a stochastic programming model to address an uncertainty-involved nurse scheduling problem that considers hospital management goals and nurse preferences. Chen et al. [22] propose a two-stage method to solve a medical personnel allocation problem in an uncertain environment. Farasat and Nikolaev [23] propose a model that relies on modern social network analysis theories to address nurse team formation and nurse shift assignment problems. Kokangul et al. [24] explore the optimization of nurse resource allocation in a neonatal intensive care unit by combining deterministic and stochastic methods, with the aim of maximizing the number of admissions while meeting constraints on key control parameters such as occupancy rate and demand satisfaction rate. Bandi and Gupta [25] address a staffing and scheduling problem for better operating room management. Turhan and Bilgen [26] and Chen and Zeng [27] propose models and algorithms to optimize nurse rostering problems. Guo and Bard [28] propose a model to address mid-term nurse scheduling considering specialized constraints, preference considerations, and overtime.

Most existing nurse resource optimization studies focus on general nursing presence and nurse scheduling problems. However, as the populations of some countries age, the traditional studies focusing on the allocation of nurse resources in various medical facilities face new challenges [29]. Some rent studies focus on analyzing the supply and demand for accompanying services, especially for elderly patients, disabled patients, and patients with complex medical conditions. Mikšová et al. [30] point out that some patients often need professionals accompanying them during their medical treatment to ensure smooth progress of the treatment process. Mutlu et al. [31] develop a co-availability scheduling model to coordinate multidisciplinary care teams to provide better healthcare services. Mohammadipour et al. [32] conduct an explanatory study on nursing presence from the perspective of inpatients and point out that nursing presence would be ideal for patient-centered care. Nilsen et al. [33] find that, in order to meet patients’ growing demand for companion services, the respective roles of nurses and general caregivers in companion services should be emphasized. Zhang [34] suggest that, during the COVID-19 pandemic, nurses became important companions to reduce the anxiety and loneliness of inpatients. Sun et al. [35] develop a computer simulation model that incorporates predictive analytics to describe the various service needs of nursing home residents and to analyze promising effective nurse staffing policies. By optimizing the staffing strategy, nurses and nursing assistants (workers) can work together to provide accompanying care services, thus improving service efficiency and quality.

However, in the literature, optimization studies for better management of accompanying services remain rare and call for more research efforts. However, although these strategies have achieved certain results, there are still many unresolved issues in the face of complex and changing medical environments and patient needs. In particular, in view of the intensification of the aging situation, more attention should be paid to optimization research of accompanying services, but there are still few studies on this in the existing literature and more research efforts are needed. This study proposes a multi-period planning model to plan dynamic nurse staffing for high-quality accompanying service supply in outpatient departments of large general hospitals and conducts a case study to verify the effectiveness of the proposed model and to generate managerial insights for better accompanying practice. Our contributions are threefold. First, a multi-period planning model is developed to optimize dynamic nurse staffing in outpatients. Second, we examine two comparison cases to highlight the benefits of our proposed model. Third, we conduct a case study to obtain managerial insights to provide better accompanying services in outpatient departments. The remainder of this article includes three sections. Section 2 presents the staffing problem formally and presents the model formulation. Section 3 conducts a case study on the West China Hospital, a large general hospital in southwestern China, to show the benefits of our proposed model and to obtain managerial insights. Section 4 draws conclusions and briefly discusses future research issues.

## 2. Problem Statement and Model Formulation

Due to dynamically fluctuating nursing demand in outpatient departments of large general hospitals, the capacity for nursing service can be wasted. Considering that both nurses and workers can provide accompanying services to outpatients and that nurses can create higher accompanying service values for outpatients, high-quality accompanying services can be better provided with an integrated dynamic staffing of nurses and workers over a multi-period planning horizon, which, however, presents huge challenges and calls for decision supports from effective planning models.

**Figure 1:**
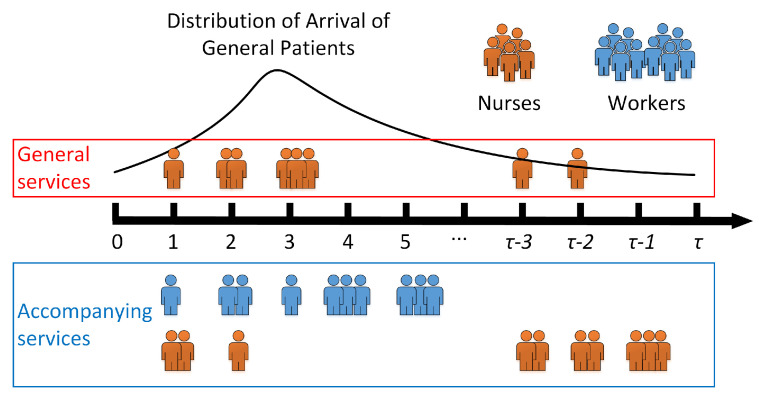
Illustration of the dynamic nurse staffing problem.

Specifically, the dynamic staffing problem maximizes the total value of the accompanying services created for outpatients without sacrificing the quality of general nursing services considering the time-varying demand for professional nursing services and accompanying services in outpatient departments. We illustrate the dynamic nurse staffing problem with Figure 1. During a planning horizon of τ periods, more general outpatients can arrive between period 2 and period 5, leading to a higher demand for general nursing services, which can only be provided by nurses, and more accompanying services are provided by workers. In contrast, in later periods from τ−3 to τ−1, the demand for general services is relatively small and more accompanying services are provided by nurses. Although the total number of nurses on duty is limited, the pool of workers is relatively large, which can effectively complement nurses to better satisfy accompanying service demand. Figure 1 shows a dynamic staffing of a limited number of nurses for general nursing services and accompanying services and illustrates coordination between nurses and workers.

Specifically, we consider a planning horizon with τ periods, which are contained in a set T={1,2,…,τ},t∈T. We let set K,k∈K include all types of professional nursing demand. We explicitly consider the dynamic general nursing demand with $P_{t}$, the number of general patients in period *t*. The total number of patients who require accompanying services in the planning horizon is indicated by *Q*, which can be obtained from the appointment system in advance. We let $\rho_{k}$ and $\phi_{k}$ be the type *k* nursing demand of a general patient and the type *k* nursing supply provided by a nurse, respectively. The total number of nurses on duty during the planning horizon is *N*. We assume that it takes π(1≤π≤τ) periods on average to provide accompanying services to a patient. The values (benefits) of providing the accompanying services to a patient by a nurse and a worker are indicated by *r* and *h*, respectively. Since in practice nurses can provide additional professional guidance to patients during accompanying services, it makes sense that r≥h. We let $g_{k}$ be the penalty cost for not providing timely type *k* professional general nursing services to patients.

Most decision variables are time-varying and indexed with a subscript *t*. We let $z_{t}\in N$ and $w_{t}\in N$, respectively, determine the number of workers and the number of nurses that begin the accompanying services in period *t*. Since the number of nurses is limited and the accompanying services last π periods on average, we let $x_{t}\in N$ find the number of nurses who still provide accompanying services in period *t*. The number of nurses who provide professional general nursing services in the period *t* is decided with $y_{t}\in N$. We employ $u_{kt}\geq 0$ to determine insufficient type *k* service capacity in period *t*. Finally, we let v∈N denote the maximum number of staff providing accompanying services in each period over the planning horizon. All notations are summarized in Table 1.

With the defined notation, the dynamic nurse staffing problem can be formulated as the following multi-period planning model (P).

(1) $$\begin{array}{cc} \left(P\right)\;\max\; & \sum_{t\in \{1,2,\ldots ,\tau -\pi \}}rw_{t}+\sum_{t\in \{1,2,\ldots ,\tau -\pi \}}hz_{t}-\sum_{k\in K}\sum_{t\in T}g_{k}u_{kt}-lv, \end{array}$$

(2) $$\begin{array}{cc} s.t.\; & x_{t}+y_{t}=N,\;\forall t\in T, \end{array}$$

(3) $$\begin{array}{cc} & u_{kt}=\max\left\{u_{kt-1}+P_{t}\rho_{k}-y_{t}\phi_{k},0\right\},\;\forall k\in K,t\in T, \end{array}$$

(4) $$\begin{array}{cc} & \sum_{t\in \{1,2,\ldots ,\tau -\pi \}}\left(z_{t}+w_{t}\right)=Q, \end{array}$$

(5) $$\begin{array}{cc} & x_{t}=\sum_{i=\{0,\ldots ,\pi -1\}:\tau -\pi \geq t-i\geq 1}w_{t-i},\;\forall t\in T, \end{array}$$

(6) $$\begin{array}{cc} & v=\max_{t\in T}\left\{x_{t}+z_{t}\right\}, \end{array}$$

(7) $$\begin{array}{cc} & z_{t}\in N,\;\forall t\in \left\{1,2,\ldots ,\tau -\pi \right\}, \end{array}$$

(8) $$\begin{array}{cc} & x_{t}\in N,\;\forall t\in T, \end{array}$$

(9) $$\begin{array}{cc} & w_{t}\in N,\;\forall t\in \left\{1,2,\ldots ,\tau -\pi \right\}, \end{array}$$

(10) $$\begin{array}{cc} & y_{t}\in N,\;\forall t\in T, \end{array}$$

(11) $$\begin{array}{cc} & v\in N, \end{array}$$

(12) $$\begin{array}{cc} & u_{kt}\geq 0,\;\forall t\in T,k\in K. \end{array}$$

The objective function (1) maximizes the total value (benefits) associated with the accompanying services over the τ-period planning horizon from the perspective of outpatients. Specifically, the first two terms are the additional total values of the provision of accompanying services by nurses and workers, respectively. The last two terms are the penalty costs associated with insufficient capacity for professional nursing service and many staff providing accompanying services simultaneously. The last penalty term is introduced considering that, when many staff provide accompanying services simultaneously, the accompanying service process can be too crowded and unpleasant for outpatients.

Constraint (2) ensures that, in each period, all nurses provide professional nursing services or accompanying services. Constraint (3) determines the insufficient capacity of the professional nursing service. Constraint (4) ensures that all accompanying service demand is met over the planning horizon. Constraint (5) determines the number of nurses providing accompanying services in each period. Constraint (6) determines *v*, the maximum number of staff providing accompanying services in a period. The other constraints, (7)–(12), define the non-negative integer variables and the non-negative continuous variables.

(P) is a mixed integer nonlinear programming model due to the nonlinear Constraints (3) and (6). However, (P) is turned into a mixed-integer linear programming (MIP) model by replacing Constraints (3) and (6) with the following constraints:

(13) $$\begin{array}{cc} u_{kt} & \geq u_{kt-1}+P_{t}\rho_{k}-y_{t}\phi_{k},\;\forall k\in K,t\in T, \end{array}$$

(14) $$\begin{array}{cc} v & \geq x_{t}+z_{t},\;\forall t\in T, \end{array}$$

where Constraint (13) and the objective function together enforce that, in the optimal solution, $u_{kt}=u_{kt-1}+P_{t}\rho_{k}-y_{t}\phi_{k}$ always holds when $u_{kt-1}+P_{t}\rho_{k}-y_{t}\phi_{k}>0$, and *v* equals to the maximum $x_{t}+z_{t}$ of all periods.

To highlight the benefits of our proposed model, we consider two comparison cases, namely (CP1) and (CP2). Specifically, (CP1) assumes that all accompanying services are provided by workers and that nurses only provide professional nursing services, which is equivalent to adding the following Constraints (15)–(17) to (P).

(15) $$\begin{array}{cc} & x_{t}=0,\;\forall t\in T, \end{array}$$

(16) $$\begin{array}{cc} & w_{t}=0,\;\forall t\in \left\{1,2,\ldots ,\tau -\pi \right\}, \end{array}$$

(17) $$\begin{array}{cc} & y_{t}=N,\;\forall t\in T. \end{array}$$

(CP2) ignores the negative impacts of letting too many nurses and workers provide accompanying services simultaneously by simply letting *l* be 0.

## 3. Case Study

This section conducts a case study on West China Hospital to show the effectiveness of our proposed model and to obtain managerial insights. We first introduce the parameter settings, then compare the optimal results of (P) and (P)s, and finally conduct sensitivity analyses on several key parameters.

### 3.1. Parameter Settings

The case study focuses on the outpatient department of West China Hospital, which needs to arrange nurse staffing within a day (a total of 8 periods with τ=8) to satisfy the general nursing demand and accompanying demand of various types of patients as much as possible. We consider three types of general nursing services, the daily nursing demand, the nursing demand of professional treatment, and the psychological support demand of patients, that is, |K|=3.

We consider a planning horizon of 8 periods, T={1,2,3,…,8}. In the planning horizon, the number of general patients who need professional nursing services $P_{t}$ changes with time. Based on our observation of the target department, the value of $P_{t}$ can be approximately set as {80,100,90,70,110,80,60,50}. The number of patients who need accompanying services during the planning horizon can be obtained from the reservation system and is set to 114 patients, that is, Q=114. The type of professional nursing demand *k* of a general patient, that is, $\rho_{k}$, should consider the relative importance and universality of each nursing demand when assigning values, and we let $\rho_{k}$ be 0.3, 0.5, and 0.4 for daily care demand, professional treatment care demand, and psychological support demand, respectively. The settings of $\phi_{k}$, the type *k* nursing supply provided by a nurse, are based on the difficulty and importance of each nursing service and are set to 1.2, 1, and 0.8 for the three types of nursing supplies, respectively. We assume that the number of nurses on duty is 50 with N=50.

We let the average number of periods π (1≤π≤τ−1) required to provide accompanying services to each patient be 2, i.e., π=2. Since nurses can provide additional professional guidance to patients during the accompanying process compared to workers, we assume that the value a nurse created by providing accompanying services to a patient is USD 25 with r=25 [36], higher than that created by a worker, which is USD 10 with h=10. We let the penalty cost for not providing type *k* general nursing services to patients in time $g_{k}$, based on the severity of the negative consequences and the characteristics of the associated demand, be USD 7, 12, and 10, respectively, for the three types of services. Finally, we let *l*, the penalty cost for the negative impacts of many staff providing accompanying services simultaneously, be USD 7.

### 3.2. Optimal Results

Considering that the size of the case study is relatively small, we apply the commercial solver Gurobi to optimally solve the model on a laptop equipped with an Intel Core 2.40 GHz i7-13700P, 16 GB RAM, and the Microsoft Windows 11 operating system. Since Gurobi is not effective in solving large instances of MIP models, we need to develop more efficient solution approaches once the case size becomes large. Based on the parameters introduced, the optimal results of (P) and (CP)s are compared in the following Table 2.

As shown, the total value of (P) is USD 982.0, which is the highest among all comparison cases. Case (CP1) only uses workers to provide accompanying services and achieves a total value USD 907.0, which is 7.6% less than that of (P). (CP2), by ignoring the potential drawbacks related to letting too many medical staff provide accompanying services simultaneously, achieves a nominal optimal value of USD 1150. However, by fixing the optimal plans of (CP2) in (P), the real optimal value of (CP2) is USD 352 (indicated in brackets), which is 64.15% less than that of (P). The optimal value comparison of the three cases showcases the effectiveness of our proposed model and highlights the importance of letting idle nurses provide accompanying services and flattening the number of medical staff providing accompanying services in each period of the planning horizon.

We illustrate the optimal dynamic staffing plan of the three cases in Table 3, Table 4 and Table 5.

The main differences between the optimal staffing plans of (P) and (CP1) are in Periods 6–8, when the general demand for nursing is relatively small. Specifically, (P) allows 5 nurses to provide accompanying services in Period 6 while (CP1) still uses workers to provide accompanying services. Such differences highlight the flexibility of nurse staffing that can be gained by applying our planning model. Although the optimal plans of (P) and (CP2) both allow nurses and workers to provide accompanying services jointly, they differ significantly in Period 1. Without considering the potential drawbacks of many staff providing accompanying services simultaneously, (CP2) satisfies all accompanying demand with workers simply in Period 1 while (P) satisfies the accompanying demand evenly in each period. This indicates that our planning model can effectively avoid overcrowded accompanying services and keep the accompanying services relatively stable over the planning horizon.

### 3.3. Sensitivity Analyses

To obtain more managerial insight, we conduct sensitivity analyses on several key parameters, including π, the average number of periods required to provide accompanying services to a patient; *Q*, the number of patients demanding accompanying services in the planning horizon; *N*, the number of nurses on duty; *r*, the value of providing accompanying services to a patient by a nurse; and $P_{t}$, the number of patients in period *t*. The sensitivity analysis results of (P) and (CP)s are compared, and the real optimal objective values of (CP2) are used.

**Figure 2:**
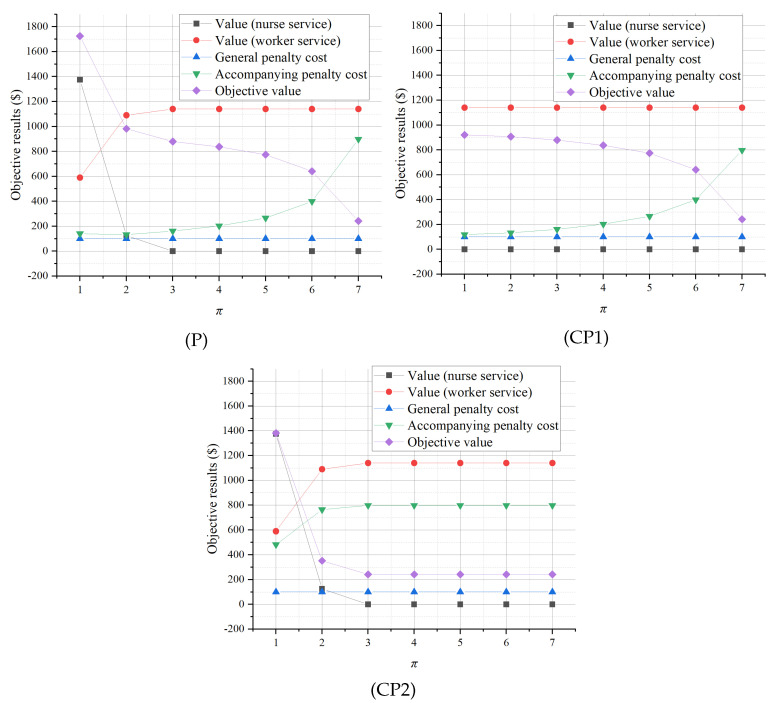
Sensitivity analysis of π.

The sensitivity analysis results on π are illustrated in Figure 2. As the period of accompanying services for each patient π increases, the optimal objective value of (P) decreases following an S-curve pattern, while those of (CP1) and (CP2) decrease concavely and convexly, respectively. This indicates that shortening the average accompanying service periods with some accompanying service training, special service procedure, and supportive policy can greatly contribute to better practical outcomes. Specifically, as π increases, the value created by the nurse (worker) accompanying services decreases (rises) to a limit in both (P) and (CP2), which shows the complementary relationship between nurses and workers and emphasizes that a shorter average accompanying period helps to obtain higher accompanying values contributed by nurses. Moreover, with the increase in π, the accompanying relevant penalty costs of (P), (CP1), and (CP2) all increase. This indicates that a longer average accompanying service time leads more staff to provide the accompanying services simultaneously, and the accompanying services became more crowded. Finally, the general penalty cost related to professional nursing services is unchanged as π increases, implying that general nursing services are not undermined by the prolonged accompanying service time.

**Figure 3:**
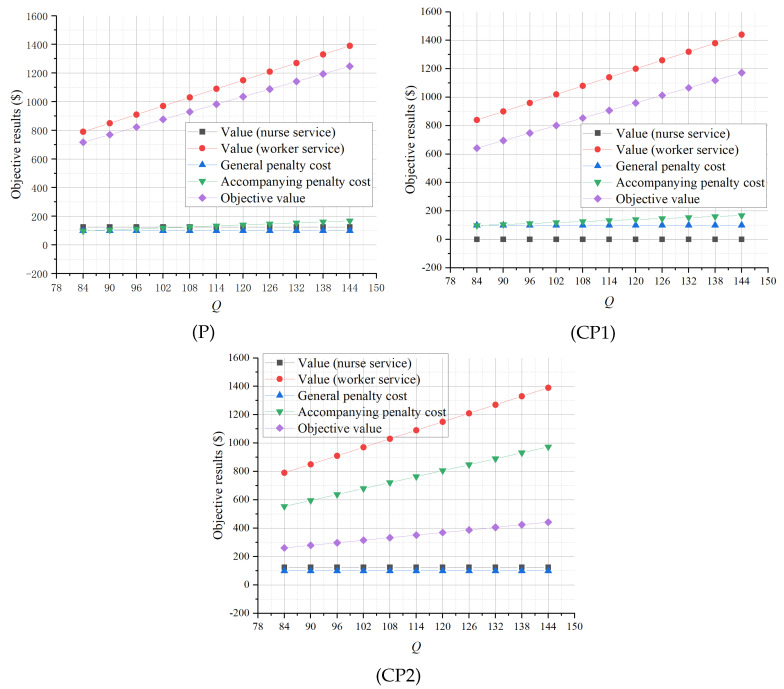
Sensitivity analysis of *Q*.

Figure 3 shows the results of the sensitivity analysis of *Q*, the number of patients who need accompanying services. As *Q* increases, the optimal objective values of (P) and (CP1) increase greatly following a similar pattern, while that of (CP2) increases slightly due to the large accompanying relevant penalty cost. This indicates that more accompanying demand can contribute to a higher accompanying service value as long as they are properly served by workers or nurses. We find that, when the number of nurses is limited and the accompanying demand increases, the additional service value is mainly created by workers, while the corresponding nurse service value and general nursing penalty cost remain stable in (P), (CP1), and (CP2). Moreover, as *Q* increases, the accompanying penalty cost of (CP2) increases rapidly, highlighting the importance of explicitly considering the negative impacts of simultaneous accompanying when the accompanying demand is high. In addition, the general penalty costs associated with professional nursing services remain unchanged in all three cases as *Q* increases, showing that our planning model can guarantee the quality of providing general nursing services by nurses when the accompanying service demand increases.

**Figure 4:**
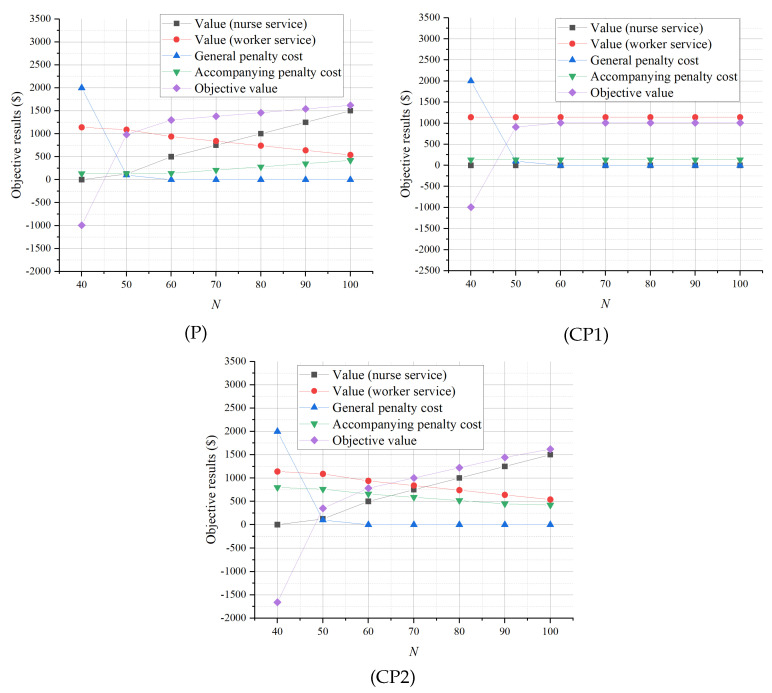
Sensitivity analysis of *N*.

The results of the sensitivity analysis of *N*, the number of nurses available, are illustrated in Figure 4. We find that the optimal objective values of (P), (CP1), and (CP2) all increase convexly up to a limit as *N* increases, and the increasing trends are more obvious with (P) and (CP2). This indicates that, when the number of nurses is small, more available nurses can help to achieve better planning results. In particular, since (CP1) does not involve nurses in the accompanying services, the objective value of (CP1) increases only when there is a shortage of nurses (that is, when N≤50) to provide general nursing services. In contrast, the increased objective values of (P) and (CP2) are attributed to the increase in the accompanying value created by more nurses and the reduction in the general nursing service penalty. This highlights that dynamically staffing nurses for accompanying services can effectively avoid waste of valuable nurse resources, especially when the number of nurses is medium or large in large general hospitals.

**Figure 5:**
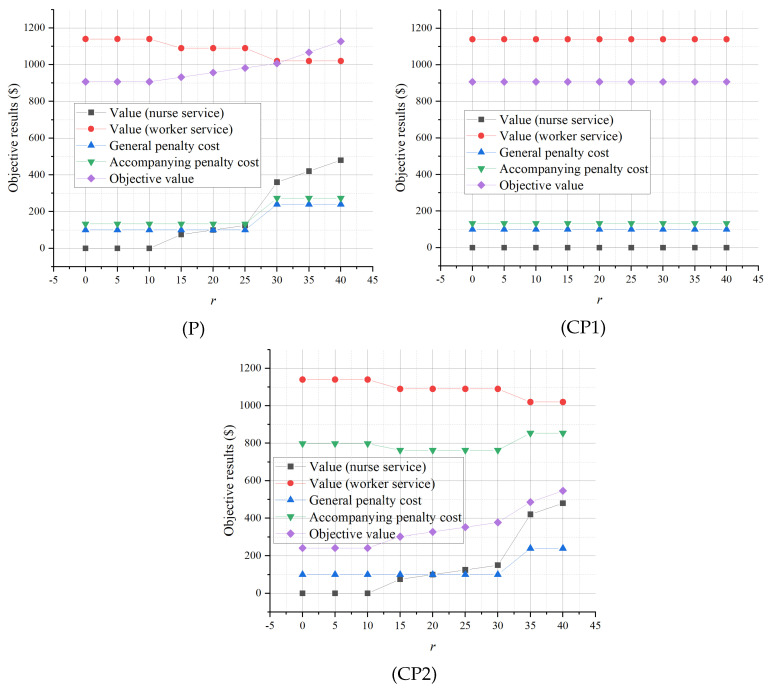
Sensitivity analysis of *r*.

The impacts of *r*, the value of a nurse providing accompanying services, are illustrated in Figure 5. Since nurses do not provide accompanying services in (CP1), *r* has no impact on the planning results of (CP1). As *r* increases, the optimal objective values of (P) and (CP2) increase, mainly due to the increased value of the accompanying services created by nurses. However, the optimal objective value of (P) is always higher than that of (CP2) due to its better control of the penalty associated with simultaneous accompanying. Moreover, we find that, as r≥30, the penalty costs of general nursing services of (P) and (CP2) increase, indicating the general nursing service can be undermined when the nurse accompanying service value is high enough. Such results emphasize that, as the value of the accompanying services of nurses increases, enabling nurses to provide accompanying services is more attractive in outpatient departments, and it becomes more important to dynamically staff nurses for general nursing services and accompanying services.

We analyze the impact of $P_{t}$, the number of general outpatients, from two perspectives: various arrival trends with the same total number and various total numbers with the same arrival trend. From the first perspective, we consider three scenarios for patient arrival. Scenario 1 assumes that patients arrive mainly in some peak periods. In this case, we let $P_{t}=\left\{40,40,200,40,40,200,40,40\right\}$. Scenario 2 keeps the total number of outpatients the same but reduces the numbers of patients in the peak period and $P_{t}=\left\{60,60,140,60,60,140,60,60\right\}$. Scenario 3 assumes that patients arrive steadily, and the number of patients arriving in each period is 80; that is, $P_{t}=\left\{80,80,80,80,80,80,80,80\right\}$. We present the results of each case in the three scenarios in the following Table 6, Table 7 and Table 8.

As shown, the optimal objective values of the three cases improve as general outpatients can display a more stable arrival pattern, which contributes to reducing the penalty cost of general nursing services caused in peak periods and making it possible for nurses to provide high-value accompanying services in some periods with low general nursing demand. Such results suggest that, when nurse resources are limited, outpatient departments should stabilize general patient arrival with more effective appointment policies and patient show-up management mechanisms, which can enhance the quality of both general nursing services and accompanying services. Moreover, in Scenario 3, Case (P) obtains the highest optimal objective value, while, in Scenario 1 and Scenario 2, the optimal objective values of (P) and (CP1) are the same. This implies that letting nurses provide high-quality accompanying services becomes more important if there are no clear peak periods in which the general nursing demand greatly exceeds the available nursing capacity.

**Figure 6:**
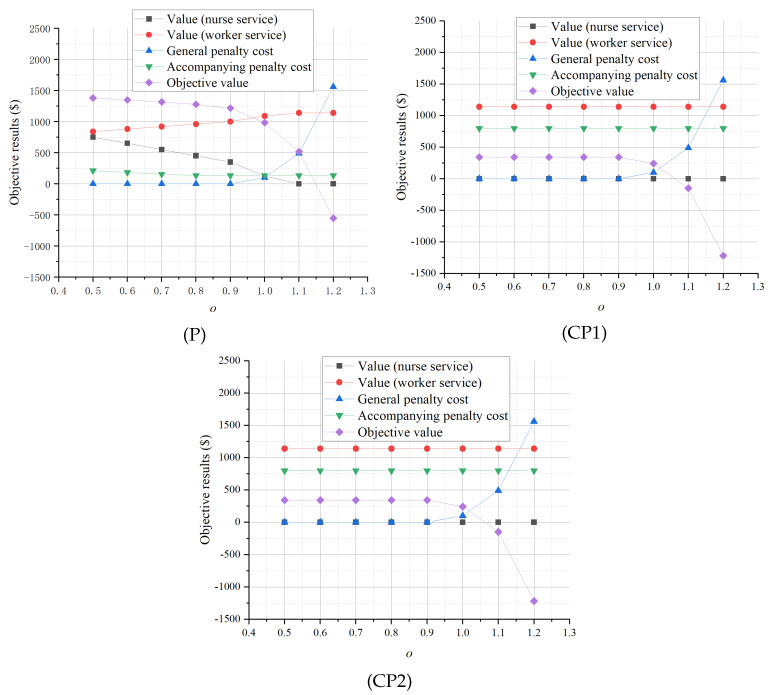
Sensitivity analysis of $P_{t}$.

From the second perspective, we keep the patient arrival trend the same as the base case setting and modify the number of patients arriving in each period with a multiplier *o*. With varying *o*, the sensitivity analysis results of $P_{t}$ are illustrated in Figure 6.

As shown, with increasing $P_{t}$, the optimal objective values of the three cases all decrease due to the fast increase in the general nursing penalty caused by limited nursing resources, and the optimal objective value of (P) is always better than those of (CP1) and (CP2). Moreover, while there is a trade-off between the accompanying service value created by workers and that by nurses in (P), such values are unchanged in (CP1) and (CP2). This not only highlights the effectiveness of (P) in dynamic utilization of nurses and workers to provide better accompanying services but also emphasizes the importance of dynamically adjusting nurse staffing according to the various patient arrival situations.

## 4. Conclusions and Future Work

This study proposed a planning model to dynamically staff nurses and workers for medical accompanying services over a planning horizon considering the dynamic arrival of various types of outpatients and a limited number of nurses. With a value maximization planning goal formulated from the perspective of outpatients, the model can effectively trade off the value created by allowing nurses and workers to provide outpatient accompanying services and the accompanying penalty costs associated with unmet accompanying demand in time and the overcrowded accompanying service. Two comparison cases were investigated to highlight the value of our multi-period planning model. With a case study, we demonstrated the effectiveness of the proposed model and gained managerial insights, such as (1) the dynamic staffing of nurses can contribute to timely and high-quality nursing services while preventing idle nurses and overcrowding, (2) dynamic nurse staffing can achieve a better cost-effectiveness balance, which improves the accompanying service value and overall efficiency, and (3) more accompanying service value can be created if nurses can efficiently serve patients with fewer periods on average, which emphasizes the importance of establishing supportive accompanying service training, procedures, and policies.

This study has some limitations, which can be addressed in the future. First, this study only considers dynamic nurse staffing and assumes that the pool of available workers is large so that workers are always available when nurses are unavailable for accompanying services. It can be important to abandon the assumption about workers and conduct integrated planning on the dynamic staffing of nurses and workers. Second, uncertainties are not considered in this study, while some general patients may require accompanying services on site without appointments and contribute to uncertain accompanying demand. A stochastic programming model or a robust optimization model can be developed to facilitate dynamic nurse staffing with uncertainties. Third, while this study focuses on nurse staffing in one department, it is interesting to investigate coordinated dynamic nurse staffing for multiple departments in a hospital. Lastly, our study does not consider operational costs and motivation mechanisms related to accompanying services. A planning model that incorporates the operational costs of the accompanying services and optimizes the salaries of nurses and workers can help to achieve a better trade-off between benefits and costs and motivate nurses and workers to provide better accompanying services.

## Figures and Tables

**Table 1:** Notations.

**Sets**
T={1,2,…,τ},t∈T, periods of a planning horizon, typically a day
K,k∈K, types of professional nursing demand
**Parameters**
$P_{t}$, number of general patients in period *t*
*Q*, number of patients who require accompanying services in the planning horizon
$\rho_{k}$, type *k* professional nursing demand of a general patient
$\phi_{k}$, type *k* professional nursing supply provided by a nurse
*N*, number of nurses on duty during the planning horizon
π, average number of periods (1≤π≤τ−1) required to provide accompanying services to a patient
*r*, value of providing accompanying services to a patient by a nurse
*h*, value of providing accompanying services to a patient by a worker
$g_{k}$, penalty cost for not providing type *k* professional nursing services to patients in time
*l*, penalty cost for the negative impacts of many staff providing accompanying services simultaneously
**Decision variables**
$z_{t}\in N$, number of workers for accompanying services in period *t*
$w_{t}\in N$, number of nurses begin accompanying services in period t∈{1,2,…,τ−π}
$x_{t}\in N$, number of nurses providing accompanying services in period *t*
$y_{t}\in N$, number of nurses providing professional nursing services in period *t*
v∈N, maximum number of staff providing accompanying services in each period of the planning horizon
$u_{kt}\geq 0$ insufficient type *k* professional nursing service capacity in period t∈{0}∪T

**Table 2:** Comparison of optimal objective values.

Case	Value (Nurse Service)	Value (Worker Service)	General Penalty Cost	Accompanying Penalty Cost	Objective Value
(P)	125.0	1090.0	100.0	133.0	982.0
(CP1)	0.0	1140.0	100.0	133.0	907.0
(CP2)	125.0 (125.0)	1090.0 (1090.0)	100.0 (100.0)	0.0 (763.0)	1150.0 (352.0)

**Table 3:** The optimal planning results of (P).

Variable	*t*							
	1	2	3	4	5	6	7	8
$z_{t}$	19	19	19	19	19	14	0	0
$w_{t}$	0	0	0	0	0	5	-	-
$x_{t}$	0	0	0	0	0	5	5	0
$y_{t}$	50	50	50	50	50	45	45	50

**Table 4:** The optimal planning results of (CP1).

Variable	*t*							
	1	2	3	4	5	6	7	8
$z_{t}$	19	19	19	19	19	19	0	0
$w_{t}$	0	0	0	0	0	0	-	-
$x_{t}$	0	0	0	0	0	0	0	0
$y_{t}$	50	50	50	50	50	50	50	50

**Table 5:** The optimal planning results of (CP2).

Variable	*t*							
	1	2	3	4	5	6	7	8
$z_{t}$	109	0	0	0	0	0	0	0
$w_{t}$	0	0	0	0	0	5	-	-
$x_{t}$	0	0	0	0	0	5	5	0
$y_{t}$	50	50	50	50	50	45	45	50

**Table 6:** Optimal objective values under Scenario 1.

Case	Value (Nurse Service)	Value (Worker Service)	General Penalty Cost	Accompanying Penalty Cost	Objective Value
(P)	0.0	1140.0	2800.0	133.0	−1793.0
(CP1)	0.0	1140.0	2800.0	133.0	−1793.0
(CP2)	0.0 (0.0)	1140.0 (1140.0)	2800.0 (2800.0)	0.0 (798.0)	−1660.0 (−2458.0)

**Table 7:** Optimal objective values under Scenario 2.

Case	Value (Nurse Service)	Value (Worker Service)	General Penalty Cost	Accompanying Penalty Cost	Objective Value
(P)	0.0	1140.0	800.0	133.0	207.0
(CP1)	0.0	1140.0	800.0	133.0	207.0
(CP2)	0.0 (0.0)	1140.0 (1140.0)	800.0 (800.0)	0.0 (798.0)	340.0 (−458.0)

**Table 8:** Optimal objective values under Scenario 3.

Case	Value (Nurse Service)	Value (Worker Service)	General Penalty Cost	Accompanying Penalty Cost	Objective Value
(P)	250.0	1040.0	0.0	133.0	1157.0
(CP1)	0.0	1140.0	0.0	133.0	1007.0
(CP2)	250.0 (250.0)	1040.0 (1040.0)	0.0 (0.0)	0.0 (728.0)	1290.0 (562.0)

## Data Availability

Data available on reasonable request from the authors.
